# Supplementary material for: A tissue-bioengineering strategy for modeling rare human kidney diseases in vivo
Source: Nat Commun. 2021 Nov 11;12:6496. doi: 10.1038/s41467-021-26596-y (PMC8586030; doi:10.1038/s41467-021-26596-y)
Supplement: Supplementary file 1 — Supplementary Information [file 41467_2021_26596_MOESM1_ESM.pdf]

Supplementary Information

A Tissue-Bioengineering Strategy for Modeling Rare Human Kidney Diseases *In Vivo*

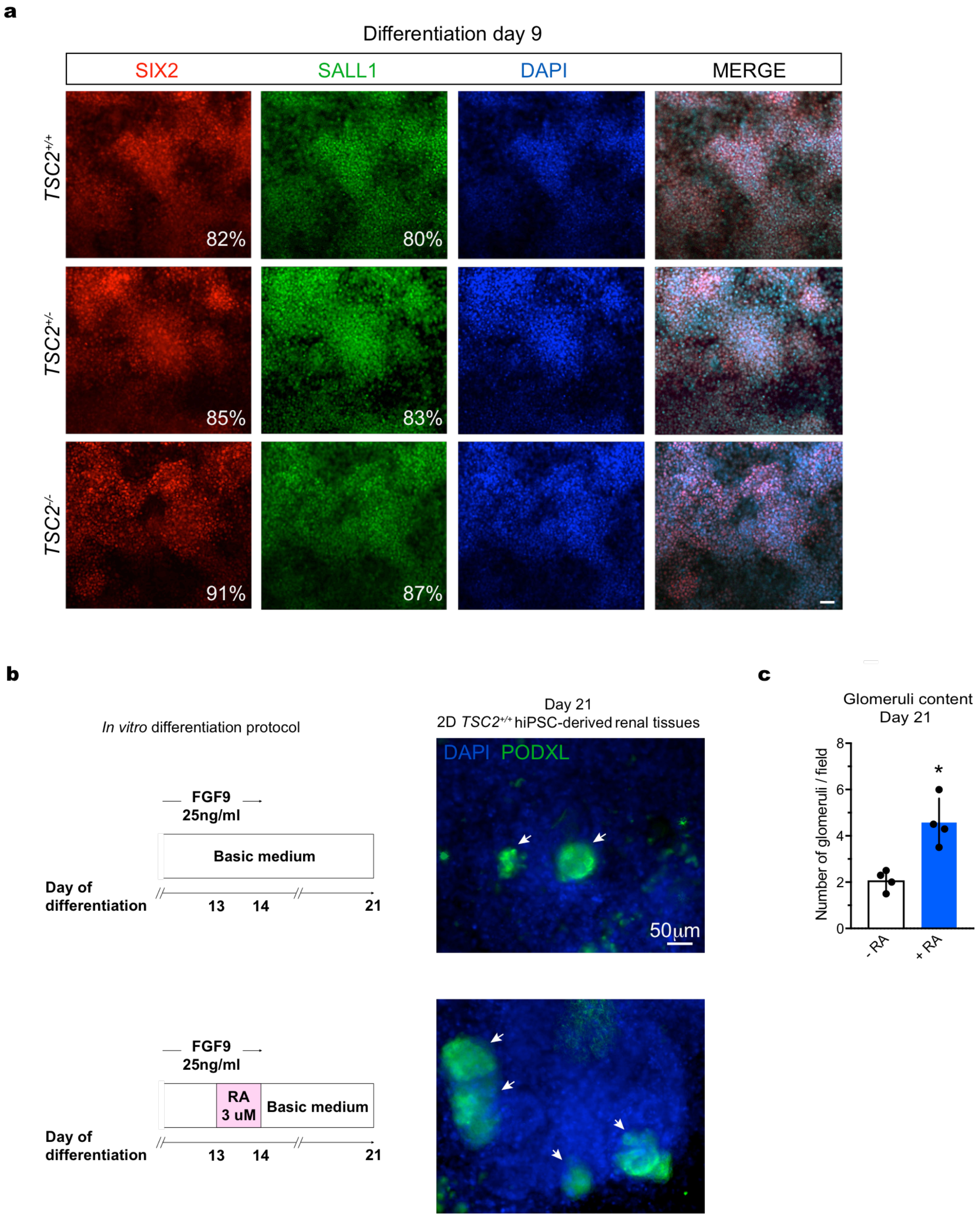

Supplementary figure 1. Nephric differentiation of isogenic *TSC2*$^{+/+}$, *TSC2*$^{+/-}$ and *TSC2*$^{-/-}$ iPSCs. a Representative immunofluorescence images showing SIX2 and SALL1 expression in 2-D *TSC2*$^{+/+}$, *TSC2*$^{-/-}$ and *TSC2*$^{+/-}$ cell cultures on Day 9 of differentiation. The percentages of positive cells quantified are indicated, the experiment was repeated four times. **b** Differentiation schematic and representative immunofluorescence images showing the effect of a 24-h retinoic acid pulse on Day 13 on formation of glomeruli containing PODXL^{+} cells. White arrows indicate glomeruli. **c** Quantification of the number of glomeruli *per* field recorded with the microscope (20X). Bar graph shows mean ± SD, n = 4 experiments of three wells each. **P*<0.05. Scale bar 50μm.

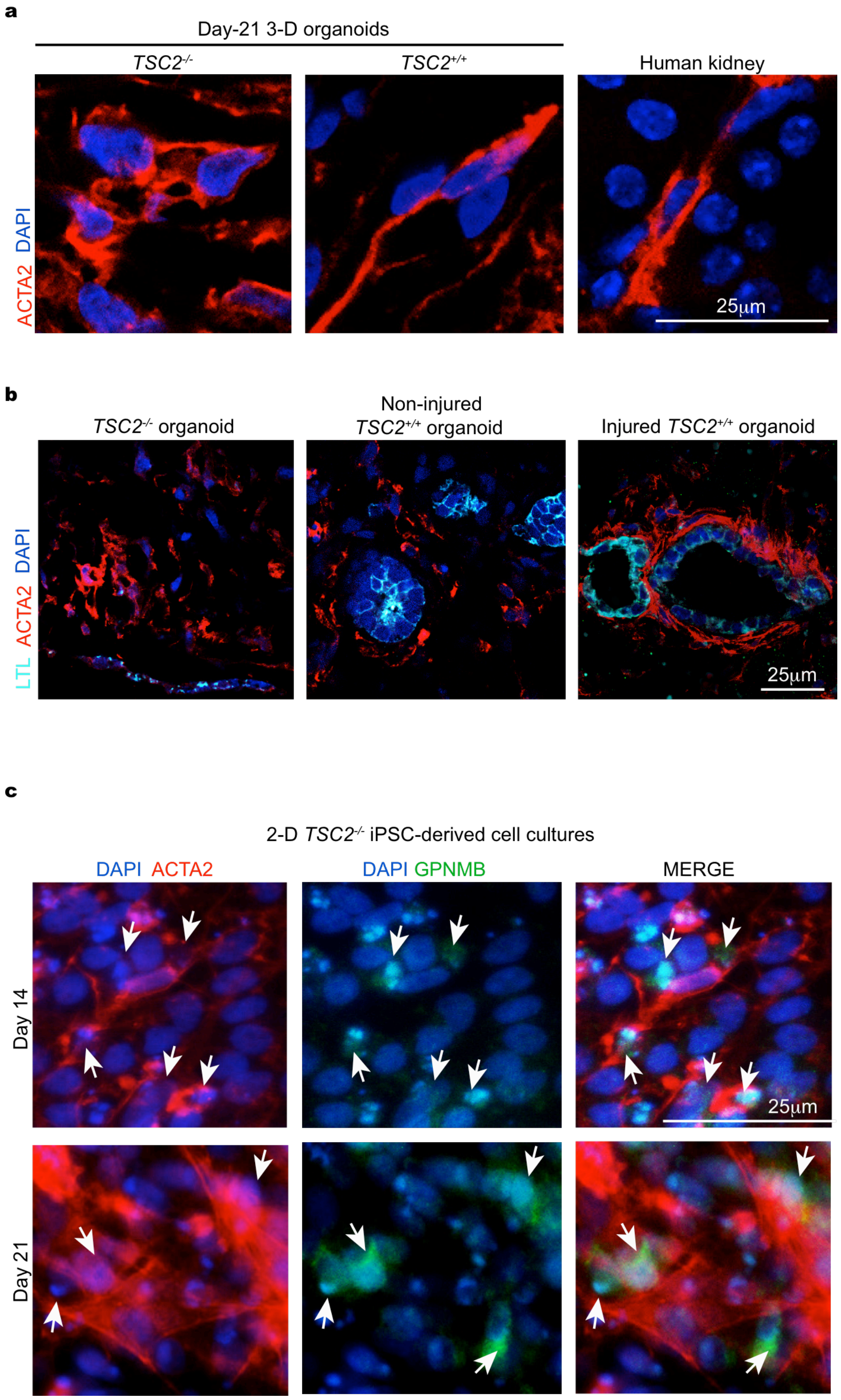

Supplementary figure 2. Identification of *TSC2*$^{-/-}$ iPSC-derived myomelanocytic cells. a Morphological appearance of AML cells in *TSC2*$^{-/-}$ renal organoids compared to *TSC2*$^{+/+}$ renal organoid and kidney fibroblasts. **b** Scattered distribution of AML cells compared to the peritubular organization of fibroblasts in normal and fibrotic *TSC2*$^{+/+}$ renal organoids. Fibrosis was induced by incubation with IL1β for 96h. Scale bars 25μm. **c** Representative immunofluorescence staining of 2D *TSC2*$^{-/-}$ iPSC derived cell cultures showing GPNMB expression in ACTA2^{+} cells on Days 14 and 21 of the nephric differentiation protocol. White arrows indicate ACTA2^{+} GPNMB^{+} cells. Scale bar 25μm.

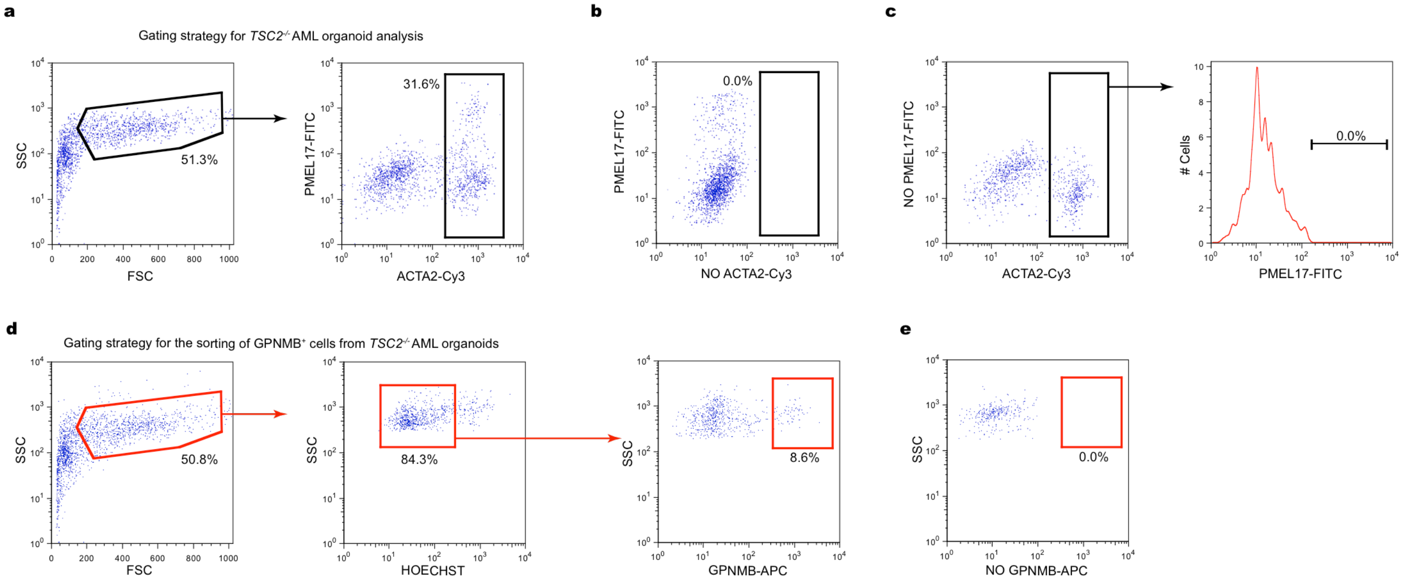

Supplementary figure 3. Flow cytometry gating strategies for the analysis and isolation of AML-like cells from *TSC2*$^{-/-}$ iPSC-derived renal organoids. **a** Flow cytometry gating strategy used to identify ACTA2^{+} PMEL^{+} cells in *TSC2*$^{-/-}$ renal organoids in figure 2e. **b**, **c** Single color controls lacking anti-ACTA2-Cy3 antibody (**b**), and anti-PMEL antibody (**c**). **d** Cell sorting gating strategy used to prospectively purify GPNMB^{+} and GPNMB^{-} cell fractions from *TSC2*$^{-/-}$ renal organoids in figure 2f. **e** Control sample lacking anti-GPNMB-APC antibody. In all samples shown, the numbers adjacent to the gates indicate representative cell frequencies.

a

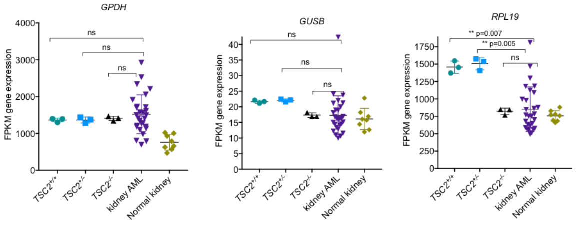

b

NAME	ES	NES	NOM p-val	FDR q-val	FWER p-val
HALLMARK_REACTIVE_OXYGEN_SPECIES_PATHWAY	0.39871022	1.7069229	0	0.05499997	0
HALLMARK_COMPLEMENT	0.59225315	1.5513009	0	0.10523207	0.137
HALLMARK_ANGIOGENESIS	0.58937246	1.4004242	0	0.14682235	0.363
HALLMARK_XENOBIOTIC_METABOLISM	0.4329361	1.3941399	0	0.15006849	0.363
HALLMARK_KRAS_SIGNALING_UP	0.47650862	1.4073737	0	0.150379	0.363
HALLMARK_INFLAMMATORY_RESPONSE	0.4672174	1.3784195	0	0.15125427	0.465
HALLMARK_ALLOGRAFT_REJECTION	0.5906101	1.466401	0	0.1587153	0.196
HALLMARK_ESTROGEN_RESPONSE_LATE	0.3445101	1.3827776	0	0.16000482	0.465
HALLMARK_INTERFERON_GAMMA_RESPONSE	0.49505466	1.3540543	0	0.1606237	0.465
HALLMARK_PEROXISOME	0.2900785	1.4142873	0	0.16400446	0.363
HALLMARK_TNFA_SIGNALING_VIA_NFKB	0.3182069	1.3261827	0	0.1682529	0.51
HALLMARK_INTERFERON_ALPHA_RESPONSE	0.48019886	1.3278441	0	0.17634226	0.51
HALLMARK_IL2_STAT5_SIGNALING	0.35907206	1.3062559	0	0.18003435	0.602
HALLMARK_APICAL_JUNCTION	0.36009854	1.3188215	0	0.18120329	0.602
HALLMARK_COAGULATION	0.64518267	1.424169	0	0.18217193	0.363
HALLMARK_FATTY_ACID_METABOLISM	0.38412678	1.2796404	0	0.1853368	0.646
HALLMARK_IL6_JAK_STAT3_SIGNALING	0.6289876	1.4391141	0	0.18589109	0.251
HALLMARK_BILE_ACID_METABOLISM	0.37456596	1.4672211	0	0.19328701	0.196
HALLMARK_APOPTOSIS	0.27065846	1.226526	0	0.20427474	0.742
HALLMARK_ADIPOGENESIS	0.26161525	1.2311289	0.10412574	0.20908016	0.742
HALLMARK_UV_RESPONSE_DN	0.28318453	1.239677	0.22857143	0.21439168	0.742
HALLMARK_EPITHELIAL_MESENCHYMAL_TRANSITION	0.41336355	1.1974243	0	0.21484017	0.786

c

NAME	ES	NES	NOM p-val	FDR q-val	FWER p-val
HALLMARK_REACTIVE_OXYGEN_SPECIES_PATHWAY	0.39871022	1.6877129	0	0.05399998	0
HALLMARK_COMPLEMENT	0.59225315	1.5637503	0	0.12149391	0.168
HALLMARK_ANGIOGENESIS	0.58937246	1.4073582	0	0.13223304	0.305
HALLMARK_XENOBIOTIC_METABOLISM	0.4329361	1.4113219	0	0.13340722	0.305
HALLMARK_PEROXISOME	0.2900785	1.4120195	0	0.14333321	0.305
HALLMARK_INTERFERON_GAMMA_RESPONSE	0.49505466	1.3601393	0	0.14820744	0.459
HALLMARK_ESTROGEN_RESPONSE_LATE	0.3445101	1.3781137	0	0.14829785	0.459
HALLMARK_INFLAMMATORY_RESPONSE	0.4672174	1.3823795	0	0.14841431	0.459
HALLMARK_COAGULATION	0.64518267	1.4263659	0	0.15324047	0.257
HALLMARK_KRAS_SIGNALING_UP	0.47650862	1.4144634	0	0.15609506	0.305
HALLMARK_ALLOGRAFT_REJECTION	0.5906101	1.4698694	0	0.15863593	0.214
HALLMARK_TNFA_SIGNALING_VIA_NFKB	0.3182069	1.3359405	0	0.16024484	0.504
HALLMARK_IL2_STAT5_SIGNALING	0.35907206	1.3176168	0	0.16463935	0.504
HALLMARK_IL6_JAK_STAT3_SIGNALING	0.6289876	1.444351	0	0.16477522	0.214
HALLMARK_INTERFERON_ALPHA_RESPONSE	0.48019886	1.3389647	0	0.1678337	0.504
HALLMARK_APICAL_JUNCTION	0.36009854	1.3079895	0	0.17421544	0.558
HALLMARK_FATTY_ACID_METABOLISM	0.38412678	1.2886324	0	0.17870884	0.66
HALLMARK_UV_RESPONSE_DN	0.28318453	1.269903	0.19842829	0.17953376	0.66
HALLMARK_BILE_ACID_METABOLISM	0.37456596	1.4786377	0	0.19351456	0.214
HALLMARK_ADIPOGENESIS	0.26161525	1.2390532	0.10136452	0.19938481	0.709
HALLMARK_EPITHELIAL_MESENCHYMAL_TRANSITION	0.41336355	1.2075171	0	0.20924948	0.759
HALLMARK_APOPTOSIS	0.27065846	1.2129807	0	0.21453014	0.759

d

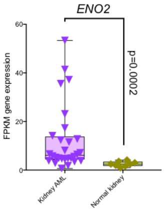

Supplementary figure 4. Transcriptional profile of *TSC2*$^{+/+}$, *TSC2*$^{+/-}$ and *TSC2*$^{-/-}$ iPSC-derived renal organoids. a Expression of housekeeping genes in RNAseq samples of *TSC2*$^{-/-}$, *TSC2*$^{+/+}$ and *TSC2*$^{+/-}$ renal organoids (n=3 each) compared to human kidney AML (n=28) and human kidney (n=8). Scatter plots display mean values ± SD. P values for individual comparisons done using a two-tailed Mann–Whitney U test are indicated. **b**, **c** Tables showing hallmark gene pathways identified by GSEA analysis of *TSC2*$^{+/+}$ *vs. TSC2*$^{-/-}$ (**b**) and *TSC2*$^{-/+}$ *vs. TSC2*$^{-/-}$ (**c**) renal organoid gene expression comparisons. **d** Expression of *ENO2* mRNA in kidney angiomyolipomas and normal kidney. Comparative mRNA expression levels for *ENO2* mRNA in human kidney AML (n=28) and human normal kidney (n=8). Gene expression is shown in FPKM values. Box-and-whisker plot showing minimum value, first quartile, median, third quartile and maximum value. P values for individual comparisons done using a two-tailed Mann–Whitney U test are indicated.

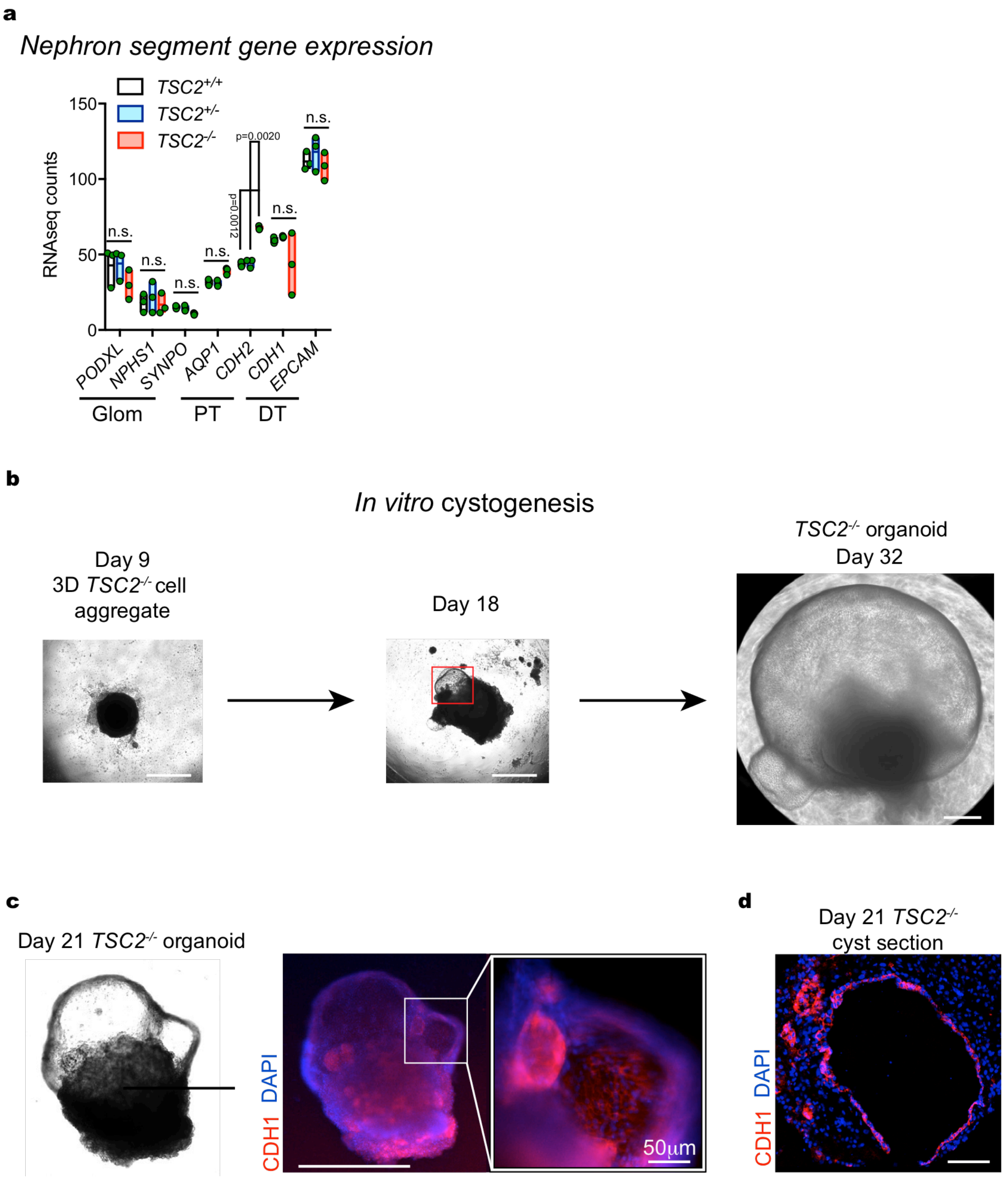

Supplementary figure 5. Cystic phenotype in *TSC2*$^{-/-}$ iPSC-derived renal organoids. a Box plot showing RNAseq expression data for nephron segment genes in *TSC2*$^{-/-}$, *TSC2*$^{+/+}$ and *TSC2*$^{+/-}$ renal organoids (n=3, 5 organoids *per* sample). P values determined by two-way ANOVA analysis using Tukey test for multiple comparisons are indicated. **b** Representative brightfield images showing *TSC2*$^{-/-}$ renal organoid cyst growth. Scale bars, 500μm. **c** Representative whole mount immunofluorescence showing the presence of cysts lined by CDH1-expressing TECs in *TSC2*$^{-/-}$ renal organoids. **d** Representative confocal micrograph showing CDH1^{+} cyst lining cells. Scale bar, 50μm.

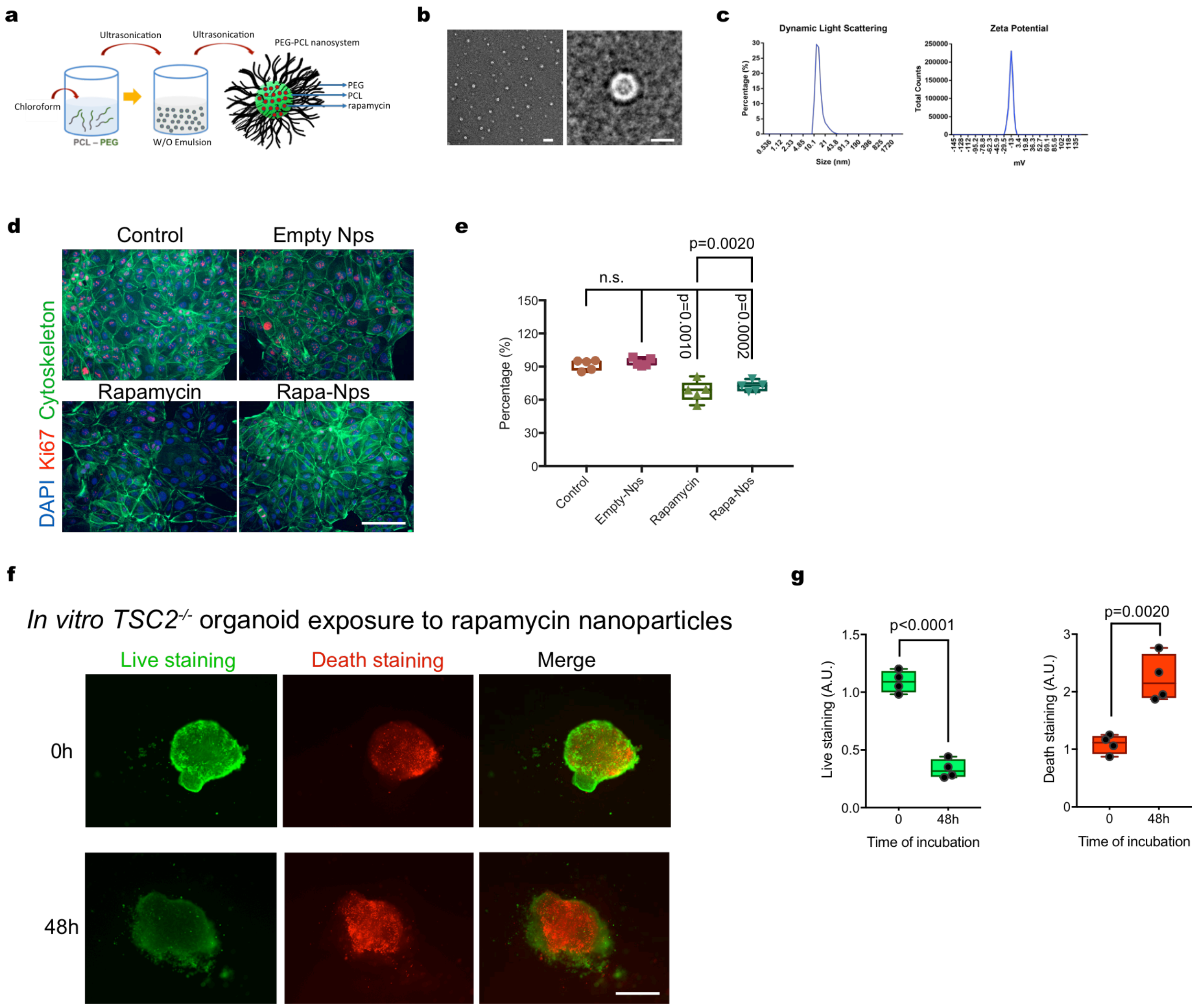

Supplementary figure 6. Fabrication of rapamycin-loaded nanoparticles. a Schematic representation of the process of Rapa-nanoparticle fabrication. **b** Representative visualization of nanoparticle morphology using transmission electron microscopy. Scale bars 10 nm. **c** TEM micrograph analysis showing the symmetric spherical shape and narrow size distribution of 12 nm with no sign of bulk participations for the Rapamycin-loaded PEG-PCL nanoparticles. **d** Representative immunofluorescence microscopy images showing Ki67 in primary mouse TECs incubated with control DMEM medium + 5% FBS (Control), DMEM medium + empty Nps (Empty-Nps), DMEM medium + rapamycin alone (Rapamycin), or DMEM medium + Rapa-Nps (Rapa-Nps). Scale bar 25µm. **e** Quantification of Ki67 expression. Box-and-whisker plot showing minimum value, first quartile, median, third quartile and maximum value. n = 5. Two independent experiments were performed with similar results. P values for individual comparisons done using two-tailed Student's t test are indicated. **f** Representative fluorescence imaging showing the viability of Day-21 *TSC2*$^{-/-}$ renal organoids exposed to rapamycin nanoparticles, at 0h and 48h of incubation. The Live cell staining is shown in the green channel, while the Dead cell staining is shown in the red channel. Scale bar, 500µm. **g** Quantification of Live cell and Dead cell signal at both time points studied. Box-and-whisker plot showing minimum value, first quartile, median, third quartile and maximum value. P values for individual comparisons done using two-tailed Student's t test are indicated. n = 4 organoids *per* treatment. Two independent experiments were performed with similar results.

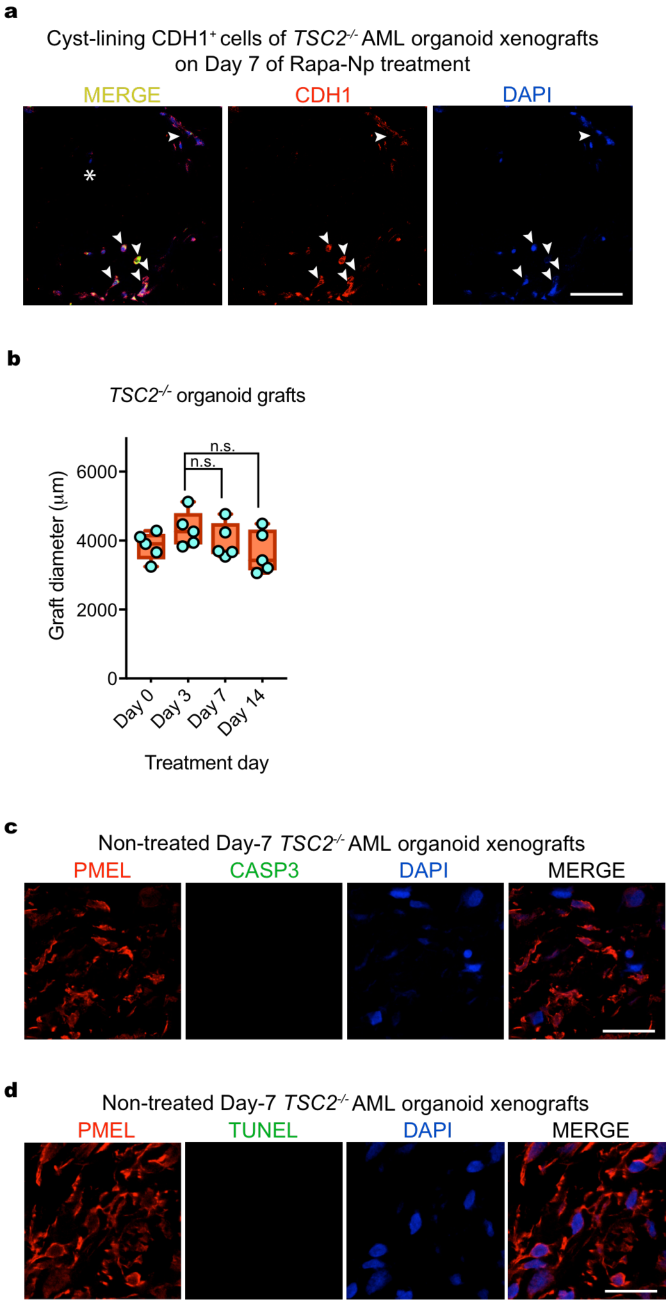

Supplementary figure 7. Effect of rapamycin treatment on transplanted *TSC2*$^{-/-}$ AML organoids. a Representative immunofluorescence images of *TSC2*$^{-/-}$ AML organoid xenograft CDH1^{+} tubule cells on Day 3 post delivery of Rapa-Nps. **b** Quantified *TSC2*$^{-/-}$ organoid graft diameter values are presented as a box-and-whisker plot showing minimum value, first quartile, median, third quartile and maximum value, n = 5 grafts. Non-significant mean differences determined by two-way ANOVA analysis using Tukey test for multiple comparisons are indicated as n.s. **c** Representative immunofluorescence images for the detection of activated Casp3 in *TSC2*$^{-/-}$ AML organoid xenograft PMEL^{+} myoid cells on Day 7 of control non-treated *TSC2*$^{-/-}$ organoid grafts. **d** Representative confocal imaging depicting detection of DNA fragmentation by TUNEL on sections of *TSC2*$^{-/-}$ AML xenografts, on Day 7 of control non-treated *TSC2*$^{-/-}$ organoid grafts. Scale bars 50μm (**a**), 25μm (**c, d**).

Supplementary Tables.

Supplementary Table 1. Pairwise differential gene expression analyses in *TSC2*$^{-/-}$ kidney organoids compared to *TSC2*$^{+/+}$ and *TSC2*$^{+/-}$ kidney organoids. Numbers of all upregulated/downregulated genes and transcription factors for adjusted p-value/FDR<0.05 and $\log_2$ fold >2/$\log_2$ fold <-2 for expression FPKM values are presented. DEseq2 analyses of *TSC2*$^{+/+}$ and *TSC2+/-* kidney organoids did not reveal significant differences in transcriptomic level (data not shown).

Differential gene expression DESeq2 analysis	*TSC2 -/-* vs *TSC2 +/+* kidney organoids		*TSC2 -/-* vs *TSC2 +/-* kidney organoids	
All DEGs (FDR<0.05 and $\log_2$>2/$\log_2$<-2)	477 ($\log_2$ >2)	362 ($\log_2$<-2)	480 ($\log_2$ >2)	363 ($\log_2$<-2)
All TFs FDR<0.05 and $\log_2$>2/$\log_2$<-2)	59 ($\log_2$ >2)	30 ($\log_2$<-2)	60 ($\log_2$ >2)	31 ($\log_2$<-2)

Supplementary Table 2. List of Taqman RT-PCR primers used.

Taqman Assay ID	Human Gene	Catalog #
Hs00232731_m1	*SIX2*	4331182
Hs04285637_m1	*SALL1*	4331182
Hs01015256_g1	*PAX8*	4351372
Hs00232144_m1	*LHX1*	4331182
Hs01001602_m1	*HNF1B*	4331182
Hs01103751_m1	*WT1*	4331182
Hs03023943_g1	*ACTB*	4331182
Hs00173854_m1	*PMEL*	4331182
Hs00194133_m1	*MLANA*	4331182
Hs01095669_m1	*GPNMB*	4331182
Hs00166156_m1	*CTSK*	4331182
Hs00426835_g1	*ACTA2*	4331182
