# Supplementary material for: A tissue-bioengineering strategy for modeling rare human kidney diseases in vivo
Source: Nat Commun. 2021 Nov 11;12:6496. doi: 10.1038/s41467-021-26596-y (PMC8586030; doi:10.1038/s41467-021-26596-y)
Supplement: Supplementary file 3 — Reporting Summary [file 41467_2021_26596_MOESM3_ESM.pdf]

nature research

Corresponding author(s): Dario R. Lemos

Last updated by author(s): 09/19/2021

Reporting Summary

Nature Research wishes to improve the reproducibility of the work that we publish. This form provides structure for consistency and transparency in reporting. For further information on Nature Research policies, see our Editorial Policies and the Editorial Policy Checklist.

Statistics

For all statistical analyses, confirm that the following items are present in the figure legend, table legend, main text, or Methods section.

n/a	Confirmed	
☐	☒	The exact sample size (*n*) for each experimental group/condition, given as a discrete number and unit of measurement
☐	☒	A statement on whether measurements were taken from distinct samples or whether the same sample was measured repeatedly
☐	☒	The statistical test(s) used AND whether they are one- or two-sided<br>*Only common tests should be described solely by name; describe more complex techniques in the Methods section.*
☒	☐	A description of all covariates tested
☒	☐	A description of any assumptions or corrections, such as tests of normality and adjustment for multiple comparisons
☐	☒	A full description of the statistical parameters including central tendency (e.g. means) or other basic estimates (e.g. regression coefficient) AND variation (e.g. standard deviation) or associated estimates of uncertainty (e.g. confidence intervals)
☐	☒	For null hypothesis testing, the test statistic (e.g. *F*, *t*, *r*) with confidence intervals, effect sizes, degrees of freedom and *P* value noted<br>*Give P values as exact values whenever suitable.*
☒	☐	For Bayesian analysis, information on the choice of priors and Markov chain Monte Carlo settings
☒	☐	For hierarchical and complex designs, identification of the appropriate level for tests and full reporting of outcomes
☒	☐	Estimates of effect sizes (e.g. Cohen's *d*, Pearson's *r*), indicating how they were calculated

Our web collection on statistics for biologists contains articles on many of the points above.

Software and code

Policy information about availability of computer code

Data collection	Raw Illumina output was converted to Fastq format using Illumina Bcl2fastq v2.18
Data analysis	Data were analyzed using ImageJ 1.51W, GraphPad Prism v7.0 and Qlucore v3.6., FlowJo v10.4.1

For manuscripts utilizing custom algorithms or software that are central to the research but not yet described in published literature, software must be made available to editors and reviewers. We strongly encourage code deposition in a community repository (e.g. GitHub). See the Nature Research guidelines for submitting code & software for further information.

Data

Policy information about availability of data

All manuscripts must include a data availability statement. This statement should provide the following information, where applicable:

- Accession codes, unique identifiers, or web links for publicly available datasets
- A list of figures that have associated raw data
- A description of any restrictions on data availability

The RNASeq datasets are publicly available under GEO series record GSE171474. All the data supporting the findings of the study are available in this article and its supplementary information files. All other relevant data are available from the corresponding author on request. The source data are provided as a Source Data file.

Field-specific reporting

Life sciences study design

All studies must disclose on these points even when the disclosure is negative.

Sample size	For the in vitro experiments, sample size was determined based on known biological variance and statistical feasibility, determined in previous studies, including Lemos DR et. al J Am Soc Nephrol. 2018 Jun;29(6):1690-1705 and Morizane R et. al Nat Biotechnol. 2015 Nov;33 (11):1193-200; and based on the variability observed for each particular experiment (e.g. larger sample size was used in experiments where more variability was expected). For in vivo experiments, group size was decided based on power calculations to achieve an alpha = 0.05 and beta<0.2 (power>80%), whilst minimising animal excess.
Data exclusions	No data were excluded in the analyses.
Replication	All experiments were replicated at least 3 times. All attempts at replication were successful.
Randomization	Randomisation was not applicable in our in vitro studies. In transplantation experiments, the animals were randomly assigned to the experimental groups irrespective of body weight or any other variable.
Blinding	In RNAseq experiments and in animal experiments the investigators were blinded to group allocation and data analysis. Investigators knew only about ID of each sample/mouse. No blinding methods were used in in vitro experiments given that organoid phenotypes were clearly visible, preventing the concealment of sample genotype.

Reporting for specific materials, systems and methods

We require information from authors about some types of materials, experimental systems and methods used in many studies. Here, indicate whether each material, system or method listed is relevant to your study. If you are not sure if a list item applies to your research, read the appropriate section before selecting a response.

Materials & experimental systems

n/a	Involved in the study	
☐	☒	Antibodies
☐	☒	Eukaryotic cell lines
☒	☐	Palaeontology and archaeology
☐	☒	Animals and other organisms
☐	☒	Human research participants
☒	☐	Clinical data
☒	☐	Dual use research of concern

Methods

n/a	Involved in the study	
☒	☐	ChIP-seq
☐	☒	Flow cytometry
☒	☐	MRI-based neuroimaging

Antibodies

Antibodies used	The following antibodies from Cell Signaling Technology were used: anti-ribosomal protein S6 (#2217,clone 5G10, 1:1000), anti-phospho S6 (#2211, 1:1000), anti-phospho P70S6K (#9205, 1:1000), anti-P70S6K (#9202, 1:2000), anti-β Actin (#4967, 1:1000), anti-GPNMB (#38313, clone E4D7P, 1:700). Anti-ACTA2 was from Sigma-Aldrich (#F3777, clone 1A4, 1:2000), anti-human pro-Caspase 3 was purchased from ThermoFisher Scientific (#MA1-41163 , clone 31A893, 1:400), anti- human cleaved Caspase 3 was purchased from Abcam (ab2302, 1:200), anti-human p21CIP1 was purchased from Novus Biologicals (#AF1047, 1:400), anti-SIX2 (Proteintech, #11562-1-AP, 1:250), anti-SALL1 (Abcam #ab41974, clone K9814, 1:100), anti-PAX8 (Proteintech, #10336-1-AP, 1:250), anti-PMEL (Agilent Technologies, #M063429-2, clone HMB-45, 1:200), anti-ACTA2 (Sigma-Aldrich #C6198, clone 1A4, 1:400), anti-GPNMB (Cell Signaling Technologies #38313, clone E4D7P, 1:300), anti-PODXL1 (R&D Systems, #AF1658, 1:400), anti-CDH1 (Abcam # AB40772, clone EP700Y, 1:400), anti-human Lamin A/C (Abcam, #ab108595, clone EPR4100, 1:300), anti-PLVAP (Univ. of Iowa, MECA32, clone SP2/0, 1:200), anti-phospho S6 (CST #2211, 1:400), anti-Ki67 (ThermoFisher Scientific, # 701198, clone 12H15 L5, 1:250), anti- human cleaved Caspase 3 (Cell Signaling Technology #9661, 1:300)
Validation	Manufacturer validations as stated on the product websites were accepted and were further confirmed against the known biology, clinical diagnostic data and performance of the antibody for example against experimental controls.

Eukaryotic cell lines

Policy information about cell lines

Cell line source(s)	The hiPSC cell lines 77-patient, 77-TSC2-null and control 77-TSC2WT were a kind gift from Dr. Mustafa Sahin.
Authentication	The cell lines were authenticated by means of western blot and gene expression analysis (RNAseq).
Mycoplasma contamination	The cell lines tested negative for mycoplasma prior to initiation of this study.

Commonly misidentified lines (See ICLAC register)	None used

Animals and other organisms

Policy information about studies involving animals; ARRIVE guidelines recommended for reporting animal research

Laboratory animals	As stated in the Materials and Methods section, ten-week-old male NIH-Foxn1rnu immunodeficient nude rats were purchased from Charles River Laboratories
Wild animals	None used
Field-collected samples	None used
Ethics oversight	All experiments were performed under protocols approved by the Institutional Animal Care and Use Committee at Brigham and Women's Hospital.

Note that full information on the approval of the study protocol must also be provided in the manuscript.

Human research participants

Policy information about studies involving human research participants

Population characteristics	The subjects enrolled in the original study involving derivation of iPSCs were recruited through Boston Children's Hospital, and informed consents were obtained from all participants and/or their parents as appropriate. The patient from whom the 77-patient iPSC-line was derived was diagnosed with Tuberous Sclerosis Complex, cortical tubers, epilepsy. The 18 kidney AML samples used in RNAseq experiments were discard pathologic specimens derived from patients who underwent surgical tumor resection. All tumors ohad a confirmed pathologic diagnosis of angiomyolipoma. The samples were anonymized, no information regarding gender, age, genotype past and current diagnosis was available. The patients were not undergoing rapalog treatment at the time of the resection. No kidney AML sample was excluded.
Recruitment	Patients were not actively recruited. No bias was applied in the kidney AML sample collection process. The clinical criterion for tumor resection was that tumor size posed life-threatening risk to the patient.
Ethics oversight	To derive and characterize the human iPSC lines at the Boston Children's Hospital (Boston MA, USA) the Human Subjects ethics committee Boston Children's Hospital Institutional Review Board (IRB) approved the study protocol (X09-08-0442). The protocol 2014P001446 "Discard human pathologic specimens" was approved by the Partners Human Research Committee of the Mass General Brigham hospitals.

Note that full information on the approval of the study protocol must also be provided in the manuscript.

Flow Cytometry

Plots

Confirm that:

- [x] The axis labels state the marker and fluorochrome used (e.g. CD4-FITC).
- [x] The axis scales are clearly visible. Include numbers along axes only for bottom left plot of group (a 'group' is an analysis of identical markers).
- [x] All plots are contour plots with outliers or pseudocolor plots.
- [x] A numerical value for number of cells or percentage (with statistics) is provided.

Methodology

Sample preparation	In each experiment, five organoids of each genotype were incubated with TrypLE Select (Thermo Fisher Scientific #12563011) in a shaker at 37°C for 10 min. The digestions were passed through a 40-µm cell strainer (Millipore Sigma #CLS431750), and washed with PBS. Resulting single cell suspensions were collected by centrifugation at 400g for 5 min. Intracellular stainings with anti-PMEL antibody HMB-45-FITC (Novus Biologicals #NBP2-34638F, 1:200) and with anti-ACTA2 (Sigma-Aldrich #C6198, 1:400) were performed using the PerFix-nc Kit (Beckman Coulter #B31167), for 30 min at 4 °C in supplemented PBS containing 2 mM EDTA and 2% FBS. Staining for human GPNMB (Fisher Scientific #17-983-842) was done on non-fixed live cell suspensions for 30 min at 4 °C in supplemented PBS containing 2 mM EDTA and 2% FBS.
Instrument	Becton Dickinson LSRII
Software	Stated in Materials and Methods, FlowJo
Cell population abundance	ACTA2+ PMEL+ cell population was ~7-8% of total cell population.

Gating strategy	As shown in Supplementary data, FSC and SSC gating was followed by gating on PMEL/SMA. Positive and negative cell populations were determined using fluorescence minus one (FMO) controls as shown in supplementary data.

☒ Tick this box to confirm that a figure exemplifying the gating strategy is provided in the Supplementary Information.
